# Effects of vitamin D supplementation on follicular development, gonadotropins and sex hormone concentrations, and insulin resistance in induced polycystic ovary syndrome

**DOI:** 10.4274/tjod.galenos.2019.46244

**Published:** 2019-10-10

**Authors:** Nasim Behmanesh, Ali Abedelahi, Hojjatollah Nozad Charoudeh, Alireza Alihemmati

**Affiliations:** 1 Tabriz University of Medical Sciences, Stem Cell Research Center, Tabriz, Iran; 2 Tabriz University of Medical Sciences, Department of Anatomical Sciences, Tabriz, Iran

**Keywords:** Vitamin D, polycystic, ovary, gonadotropin

## Abstract

**Objective:**

Polycystic ovary syndrome (PCOS) as a reproductive disorder disturbs ovarian follicular development, vitamin D stimulated insulin activity, and sex hormone concentrations. This study aimed to examine the effects of vitamin D on ovarian follicular development, insulin resistance, and sex hormone changes in rats with induced PCOS.

**Materials and Methods:**

Forty female Wistar rats were randomly divided into four groups: (1) control, (2) induced PCOS, (3) vitamin D-treated non-PCOS (sham group), (4) vitamin D treated PCOS groups. All rats were then sacrificed under anesthesia and ovarian tissue samples were evaluated histomorphometrically. Blood samples were collected for analyzing the serum concentrations of sex hormones and insulin resistance.

**Results:**

The number of atretic follicles at different stages of development increased in the PCOS ovaries (p<0.001). Vitamin D treatment significantly increased the normality of follicles in rats with PCOS (p<0.001). The serum concentration of follicle stimulating hormone and the estradiol significantly increased in rats with PCOS, whereas the testosterone and luteinizing hormone concentrations, glucose, insulin, and insulin resistance concentrations significantly decreased during vitamin D treatment (p<0.001).

**Conclusion:**

This study indicated that vitamin D treatment may protect ovarian tissue from the negative effect of PCOS by improving insulin activity and gonadotropin concentrations.

**PRECIS:** We have found that daily intake or injection vitamin D improves the symptoms of Polycystic ovary syndrome and also decreases body mass index and ultimately regulates and balances the sex hormones.

## Introduction

Polycystic ovary syndrome (PCOS) is a type of endocrine disorder, characterized by disturbances of androgen secretion, which can result in the disruption of cyclicity and the induction of polycystic ovaries^(1,2,3). ^These conditions can disturb and block the follicular development and induce oligo/anovulation and infertility^(3)^. The majority of the available studies show that testosterone and luteinizing hormone (LH) concentrations are higher in patients with PCOS^(4)^. Aromatase activity stimulates granulosa cells to convert androgens to estrogens, resulting in a balance between androgens and estrogen productions. In contrast, the inhibition of aromatase activity in granulosa cells suppresses the conversion of testosterone to estrogen and reduces estradiol concentrations, leading to anovulation^(5,6,7)^. Moreover, the majority of patients with PCOS are at a higher risk of obesity and insulin resistance (IR)^(8)^. Some studies have established an association between vitamin D deficiency (concentration <50 nmoL/L), obesity, IR, and infertility in patients with PCOS and have identified vitamin D deficiency as the main factor contributing to hyperandrogenism^(9,10,11)^. Vitamin D entering the body is either in the ergocalciferol (D2) or cholecalciferol (D3) form. D2 is obtained from plants, and D3 is made in the cells of the epidermis. Vitamin D is then converted to 25-hydroxyvitamin D in the liver and 1.25 dihydroxyvitamin D (calcitriol) as the active form in the kidney^(12)^. Vitamin D is a steroid hormone that regulates numerous actions, including calcium, insulin, and phosphorus metabolism in different tissues of the body^(13)^. The vitamin D receptor is expressed in the ovary, endometrium, placenta and testis, suggesting that vitamin D plays a critical role in these tissues^(14)^. Previous studies have highlighted the role of vitamin D in female reproductive functions such as steroidogenesis, which can enhance granulosa cell proliferation, oocyte activation, ovulation, and follicular development^(15,16,17)^. Parikh et al.^(18)^ demonstrated that vitamin D induced the production of progesterone, estrogen, and insulin-like growth factor-binding protein 1 in human ovarian cells. However, vitamin D deficiency is commonly found in women with PCOS, but the role of vitamin D deficiency in ovarian tissue structure and patients with PCOS is not yet entirely clear. Therefore, the aim of the current study was to investigate the effect of vitamin D on ovarian follicular morphology, follicular development, androgen concentrations, IR, and insulin activity in rats with PCOS.

## Materials and Methods

### Chemicals and experimental animals

All chemicals were purchased from Sigma-Aldrich, unless otherwise indicated. All methods and experiments were approved by The research protocol of this study was approved by Vice Chancellor for Research of Tabriz University of Medical Sciences and Ethics in Research Committee of Tabriz University of Medical Sciences, (under code number: TBZMED.REC.94/2-5/7). In this study, 40 healthy adult Wistar albino female rats aged 8 weeks and weighing 200±20 g were obtained from the Animal Care Center of Tabriz University of Medical Sciences. Rats were housed in a controlled cycle of 12 hours’ light and 12 hours’ darkness at temperatures of 24-24 °C with free access to water and food.

### Experimental design

Forty female rats were randomly assigned to the following 4 treatment groups (n=10 for each group):

-Group 1: control group, rats were not injected,

-Group 2: induced PCOS groups, rats received estradiol valerate,

-Group 3: sham groups, rats received vitamin D (vitamin D treated non-PCOS group),

-Group 4: vitamin D treated PCOS group, rats were induced by estradiol valerate and then treated with vitamin D.

## Evaluation of the sexual cycle

Estrous cycles were evaluated 1 week before the experiment and during the treatment between 8:00 a.m. and 10:00 a.m. The samples in the experimental group were examined daily^(19)^. The vaginal smears were dried and monitored under a light microscope at magnification of 400x, and then the relative frequencies of leukocytes, and cornified epithelial cells were calculated. The mice were evaluated regularly for 4-5 days for both control and experimental groups and all the study rats had a regular period before performing the experiment.

### Polycystic ovary syndrome induction model

The injection site was sterilized and 2 mg/kg body weight (BW) single dose of E.V. (Aburaihan, Iran) was injected subcutaneously for 60 days. The induction of PCOS was verified by vaginal smears and histologic and serologic examination was performed for a period of 60 days^(20)^.

### Preparation and administration of vitamin D

Vitamin D was purchased from Abu Ravihan Company (Iran) and about 2 mg of vitamin D was dissolved in dimethyl sulfoxide solution under standard conditions (away from sunlight, humidity, and microbial conditions), and stored at -20 °C. The sham group and PCOS-induced rats were injected using 1 mg/kg of vitamin D subcutaneously for 14 days at 10:00 a.m. At the end of the treatment, the ovary and BWs of the rats were measured, and then 5 mL blood was withdrawn directly from the heart of the anesthetized rats. Blood samples were placed into centrifuge tubes of 3000× g for 10 minutes and the plasma was collected and stored at -70 °C until required for hormonal analysis.

### Histopathologic observations

For histopathologic assessment, all rats were sacrificed by anesthesia and the ovaries were excised and immediately fixed in 4% (w/v) paraformaldehyde solution, dehydrated in concentrations of alcohol, cleared with xylene, embedded in paraffin wax, and tissue blocks were serially sectioned at 5 µm. The serial ovarian sections were stained with hematoxylin and eosin and viewed under a light microscope (Olympus, Japan). All follicles were classified as normal and atretic. The follicles were classified as normal if they had intact oocytes and a complete layer of granulosa cells or atretic if vacuolization and pyknotic nuclei were present in the granulosa cells and occasional shrinkage of oocytes was observed^(21)^.

### Histomorphometric analysis

The follicles were divided into the following four groups based on their developmental stages: (1) primordial follicles (oocytes of follicles surrounded by a layer of squamous or flattened granulosa cells); (2) primary follicles (oocytes surrounded by a single layer of cuboidal granulosa cells); (3) preantral follicles (oocytes surrounded by more than one layer of cuboidal granulosa cells with no antrum); and(4) antral follicles (oocytes surrounded by more than one layer of cuboidal granulosa cells with a visible antrum). The percentage of follicles at every stage per ovary was determined by counting the total number of follicles in sections. All follicles were counted when the nuclei of the oocytes were visualized and counting was repeated three times and averaged^(22)^. The number of corpora lutea (CL) and thickness of the granulosa cells, as well as the thecal cell in the ovaries of control and treated rats were evaluated.

### Ovarian follicular viability

**Figure 1:**
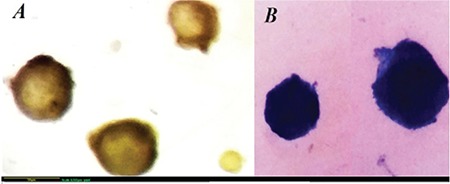
(A-B) Trypan blue staining, the follicles categorized as viable if the oocyte and surrounding granulosa cells were not stained and as degenerated follicles if stained blue

Different stages of ovarian follicles were mechanically isolated under a stereomicroscope (SZ-STS, Olympus, Tokyo, Japan) and were assessed through membrane-enclosed granulosa cells and central oocytes. Ovarian follicles were stained using 0.4% Trypan blue and detected using an inverted microscope (Olympus, Japan). The follicles were scored as viable if the oocytes and surrounding granulosa cells were stained and were assessed as degenerate follicles if the central oocytes and surrounding granulosa cells were not stained (Figure 1)^(23)^.

### Follicle stimulating hormone and luteinizing hormone and steroidal hormone measurement

The blood samples of anesthetized rats were collected and centrifuged at 3000 g for 10 min, and then the plasma of both the control and experimental groups was separated and stored at -70 °C for the measurement of follicle stimulating hormone (FSH) and LH concentrations and sex steroid hormones (such as testosterone, estrogen, and progesterone). The gonadotropin and sex hormones were measured using an enzyme-linked immunosorbent assay kit (Monobind Inc., USA) according to the manufacturer’s instructions.

### Glucose, insulin, lipid marker, and insulin resistance assays

The stored serum was used to measure glucose, insulin, and IR in the control and experimental groups. Plasma glucose and lipids concentrations were assayed using the Siemens Dimension MAX (Siemens Healthcare Diagnostics Inc.). Plasma insulin was evaluated using a magnetic affinity immunoassay (Insulin MPAIA Kit) according to the manufacturer’s instructions. The homeostasis model assessment-IR (HOMA-IR) was calculated using the formula described by Matthews et al.^(24)^.

### Statistical Analysis

To determine the effects of vitamin D supplementation on ovarian structures and androgen concentrations, we used mean ± standard deviation and one-way analysis of variance. All statistical analyses were performed using the SPSS version 16 software package. P values <0.05 were considered statistically significant.

## Results

### Body and ovary weights

The BW of rats with induced PCOS and treatment rats are shown in Table 1. In the PCOS group, both the BW and ovary weight were significantly increased by elevating abdominal fat tissue and increasing follicular fluid and ovarian stroma, respectively (p<0.001). Treatment with vitamin D in rats with PCOS for 14 days induced weight loss and significantly decreased ovarian weight compared with the non-treatment rats with PCOS (p<0.001).

### Histopathologic observations of ovarian tissue

**Figure 2:**
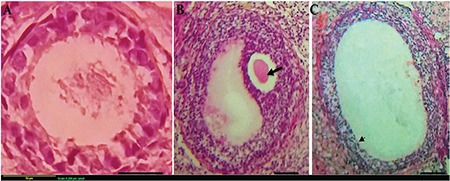
Various stages of ovarian follicles in the polycystic ovary syndrome group. a) Preantral follicles, b) antral follicles, c) atretic follicles. The number of degenerated oocytes (arrow head) increased and the thickness of granulosa cells layer decreased in the polycystic ovary syndrome group (black arrow)

**Figure 3:**
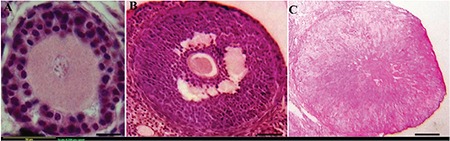
Various stages of ovarian follicles in the vitamin D-treated/ polycystic ovary syndrome group (vitamin D + polycystic ovary syndrome). a) Preantral follicles, b) Antral follicles, c) Corpus luteum. The morphology of follicles and corpora lutea were normal in vitamin D-treated rats

Histologic observation in the control and vitamin D-treated non-PCOS rats indicated that ovarian follicles at different stages of development were normal and intact. In contrast, the preantral (Figure 2a) and antral follicles (Figure 2b) showed more signs of degeneration, including granulosa cell pyknosis (Figure 2b), thin granulosa cells layer (Figure 2c), numerous atretic follicles, thickened theca layer, and absence of CL in PCOS rats (estradiol valerate-administrated group). The ovarian follicles were well developed in vitamin D-treated rats and normal granulosa cells and theca cells layer were observed (Figure 3a, b), and CL were defined (Figure 3c), indicating that the ovarian follicles were matured (folliculogenesis) and ovulated in rats treated with vitamin D.

### Histomorphometric assay of ovarian tissue

The histomorphometric analysis of ovarian follicles in the control and experimental groups is presented in Table 2. In PCOS ovaries, a significant decrease was observed in the number of follicles at the different stages of development. In PCOS rats, the percentage of normal follicles at the different stages of development was significantly decreased compared with the controls (p<0.001), whereas the number of atretic follicles in rats with PCOS was higher than in the other groups (p<0.001). In the rats with PCOS treated with vitamin D, the number of ovarian follicles at the different stages of development significantly increased and the number of normal follicles reversed as compared with the PCOS group (p<0.001). Data analysis showed that the percentage of atretic follicles at the different stages of development was significantly lower in the vitamin D-treated PCOS rats in comparison with the PCOS group (p<0.001).

### The viability of ovarian follicles

The viability of ovarian follicles isolated from the control and experimental groups is presented in Table 3. The viability of ovarian follicles at the different stages of development was significantly decreased in the PCOS group compared with the controls (p<0.001). In rats with PCOS treated with vitamin D, the viability of follicles at the different stages of development was significantly increased in comparison with the PCOS groups with no treatment (p<0.001), whereas the degenerated follicles at the different stages of development were significantly decreased (p<0.001).

### Effects of vitamin D supplementation on corpora lutea, granulosa cells, and theca layers

The number of CL, and theca and granulosa layer diameters in the control and experimental groups is presented in Table 4. The thickness of the theca layer was significantly increased in the PCOS group compared with the controls (p<0.001), whereas the thickness of granulosa layers and the number of CL were significantly decreased (p<0.001). Treatment with vitamin D significantly increased the number of CL and granulosa cells (p<0.01) but decreased the thickness of the theca layer (p<0.001).

### Effects of vitamin D supplementation on hormone concentrations in the serum of rats with polycystic ovary syndrome

The serum concentrations of gonadotropin and sex hormones in the control and experimental groups are presented in Table 4. The serum concentration of LH was significantly increased in rats with PCOS compared with the controls (p<0.05), whereas the FSH concentration was significantly decreased (p<0.05). In rats with PCOS treated with vitamin D, there was a significant decrease in LH concentration, but the FSH concentration was significantly increased (p<0.05). In rats with PCOS, testosterone concentrations were significantly increased within a few days of the end of the study (p<0.001), whereas the serum concentrations of estradiol and progesterone were significantly decreased compared with the controls (p<0.001). Treatment with vitamin D in rats with PCOS significantly decreased the testosterone concentration and increased the estradiol and progesterone concentrations (p<0.001).

### Effects of vitamin D supplementation on the serum concentrations of glucose and insulin, and insulin resistance in rats with polycystic ovary syndrome

The data of insulin, glucose, and IR (HOMA-IR) are presented in Table 4. The serum concentrations of insulin, glucose, and IR in rats PCOS were significantly higher than those in controls (p<0.001), and the insulin, glucose, and IR concentrations in vitamin D-treated PCOS group were significantly lower than those in the non-treated PCOS group (p<0.001).

### Effects of vitamin D supplementation on lipid concentrations in rats with polycystic ovary syndrome

The serum concentrations of triglyceride, cloistral, and low-density lipoprotein (LDL) in rats with PCOS were significantly higher than those in controls, whereas the concentration of high-density lipoprotein (HDL) was significantly lower (Table 4, p<0.001). In vitamin D-treated rats, a significant reduction was observed in the triglyceride, cloistral, and LDL concentrations, but there was a higher significant in the concentration of HDL compared with non-treated rats with PCOS (p<0.001).

## Discussion

The present study demonstrated that an imbalance gonadotropin hormone as a negative effect of PCOS could affect follicular development. Treatment with vitamin D decreased the adverse effects of PCOS, which is one of the most important hormone disorders, resulting in infertility for unknown reasons. The histologic observations of ovarian tissue of rats with induced PCOS demonstrated a large number of cysts and damaged follicles at the different stages of development with atrophy granulosa cells and hypertrophy theca layer. In this study, the percentage of the atretic follicles at the various stages of development decreased in rats with PCOS, and then growing follicles and folliculogenesis were impaired, and ovulation did not occur during development. Some studies found that vitamin D deficiency was associated with various PCOS symptoms, including IR , infertility, and hirsutism by gene transcription and hormonal modulation^(25,26,27)^. Wehr et al.^(26)^ reported that the patients with PCOS with hirsutism had lower vitamin D concentrations as compared with those without hirsutism (21.4 ng/mL vs. 26.8 ng/mL, respectively). Selimoglu et al.^(28)^ demonstrated that 300.000 units of vitamin D3 oral supplementation in patients with PCOS significantly decreased the HOMA-IR, but no significant changes were observed in glucose or insulin concentrations. However, in this study, vitamin D3 supplementation decreased the glucose or insulin concentrations in rats with PCOS. Our results confirmed that all rats with PCOS had higher concentrations of HOMA-IR or insulin resistant. Moreover, vitamin D was negatively correlated with IR in rats with PCOS treated with vitamin D. An increasing number of studies have found that IR is common in women with PCOS and also vitamin D deficiency is associated with IR^(29,30)^. Recent evidence suggests that vitamin D may decrease IR by stimulating the expression of insulin receptors in ovarian tissues, the renin-angiotensin-aldosterone system, and the calcium regulatory system, thereby increasing insulin sensitivity^(31,32,33)^. The results of the study of Kotsa et al.^(34)^ indicated that the majority of women with PCOS had vitamin D deficiency and abnormalities in the PTH-vitamin D axis. Many studies demonstrated that IR and obesity were associated with vitamin D deficiency and a reduction in gonadotropin hormone secretion in women with PCOS^(25,35,36)^. In the present study, the lipid profile was evaluated as an indirect index of insulin sensitivity. The analysis indicated a decrease in HOMA-IR, glucose, total cholesterol and LDL concentrations, and an increase in HDL concentrations after treatment with vitamin D in rats with PCOS. The present study highlighted the fact that vitamin D might be associated with an improvement in both insulin sensitivity and effectiveness, and with a decrease in fat mass and obesity in rats with PCOS. Treatment with vitamin D decreased the body and ovary weight in rats with induced PCOS. This could be associated with the reduction of testosterone concentrations under the influence of vitamin D. Vitamin D deficiency and hyperandrogenism are common symptoms in the initiation and development of PCOS, suggesting that inverse associations exist among vitamin D concentrations and testosterone concentrations, and vitamin D deficiency induces hyperandrogenism^(37,38)^. Our study, in agreement with Pal et al.^(39)^ demonstrated a significant decrease in testosterone concentrations in patients with PCOS after vitamin D supplementation^(39)^. Karadağ et al.^(40)^ suggested that decreased androgen concentrations might have been associated with the elevation of insulin sensitivity, and a decrease in insulin concentrations might have increased sex hormone-binding globulin concentrations and decreased the circulation of androgen concentrations. Thys-Jacobs et al.^(41)^ revealed that vitamin D deficiency induced follicular arrest and impaired folliculogenesis through calcium dysregulation, which resulted in menstrual irregularity, ovulatory, and fertility dysfunction. Another study indicated that calcium-vitamin D regulated the menstrual cycle and treated anovulation and oligomenorrhea in patients with PCOS^(42)^. In the present study, the menstrual irregularity in rats with PCOS returned to normal and regular conditions after vitamin D treatment. These results highlight the critical role of vitamin D in oogenesis and oocyte maturation. Vitamin D deficiency could be responsible for disorders of oocyte development in PCOS. First, the results of a study conducted by Patra et al.^(43)^ demonstrated a positive correlation between vitamin D deficiency and IR in PCOS. Moreover, our results showed the highest IR (HOMA-IR values) in rats with PCOS as compared with controls. Finally, vitamin D may enhance insulin activity by stimulating the expression of insulin receptors and thereby improving insulin responsiveness for glucose transport. Our results indicated that vitamin D decreased high concentrations of insulin and glucose in rats with PCOS. These results show that rats with PCOS have insufficient vitamin D and supplementation of vitamin D plays a critical role in the treatment of PCOS in rats. Studies have shown that vitamin D also has a role in gonadal steroidogenesis in both cellular and serum concentrations^(18)^. In this study, vitamin D increased estrogen concentrations by improving aromatase activity and progesterone concentrations through corpus luteum formation in rats with PCOS.

In the present study, the FSH concentrations decreased and LH concentrations increased in rats with PCOS, thus the maturation of follicles was impaired and multi-sized cystic follicles were formed. Vitamin D may induce granulosa cell differentiation by inhibiting anti-mullerian hormone expression, thereby allowing follicles to reach terminal maturation. Our results showed that the thickness of granulosa cells increased in vitamin D-treated rats with PCOS. Moreover, in luteinized granulosa cells, vitamin D resulted in granulosa cells being less dependent on FSH and more dependent on LH, thereby the matured follicles ovulated^(44)^. In addition, vitamin D induced follicular development by antiapoptotic functions and the regulation of Bcl-family and Bax^(45,46)^. Vitamin D plays an important role in estrogen and progesterone biosynthesis in the ovary. Our results were consistent with those of Kinuta et al.^(45)^, demonstrating that vitamin D promoted folliculogenesis and follicular development in rats with PCOS by increasing estrogen and progesterone concentrations and regulating the FSH and LH ratio.Vitamin D deficiency increases PTH secretion (a decrease in serum calcium concentration) because insulin plays a role in calcium absorption^(47)^. Many studies have demonstrated that the number of follicles larger than 14 mm in the calcium-vitamin D-treated group was higher than in those who received vitamin D, and this has been associated to the presence of calcium, which can increase the effect Vitamin D when used with calcium. It also regulates sex cycles and syndrome symptoms, including decreased insulin concentrations and reduced blood pressure. Calcium-vitamin D intake, which can help increase calcium and lower blood glucose concentrations, reveals the effect of calcium on the alteration of insulin secretion disorders and the increased efficacy of vitamin D^(11,3,32,41)^. Therefore, the measurement of the serum calcium concentrations and its use during treatment with vitamin D can be effective.

## Conclusion

Vitamin D supplementation in rats with PCOS regularized the androgen hormones ratio, increased insulin sensitivity, and thereby stimulated the development of the dominant follicles and the ovulation of matured follicles. Therefore, our study provides further support for the idea that vitamin D supplementation can protect ovarian tissue from the negative effects of PCOS. However, further attempts and longitudinal intervention studies are needed to evaluate the effect of vitamin D in treating PCOS models.

## Figures and Tables

**Table 1:**
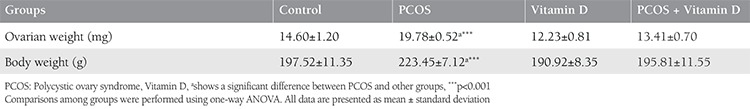
Effect of vitamin D supplementation on body weight and ovary weight in rats with polycystic ovary syndrome

**Table 2:**
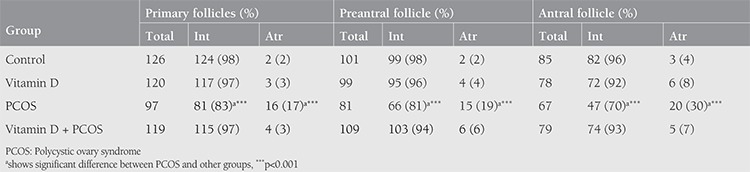
Histomorphometric assay of follicles at the various stages of development after hematoxylin-eosin staining

**Table 3:**
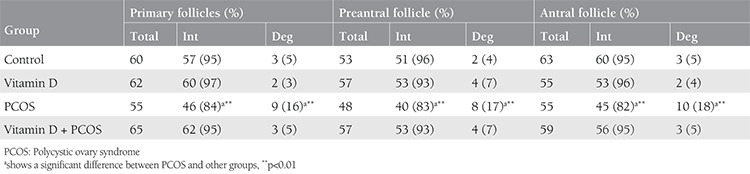
The viability of follicles at the various stages of development after Trypan blue staining

**Table 4:**
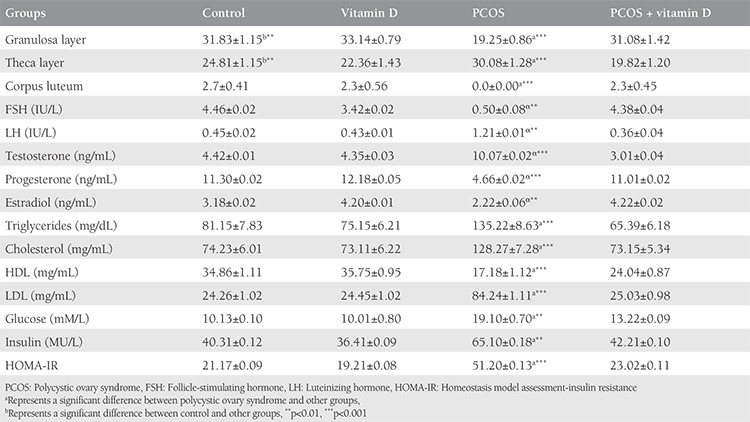
The number of corpora lutea in the ovaries, the thickness of the granulosa layer (mm), the theca layer (mm) in the antral follicles, the serum concentrations of sex steroids and lipid markers, glucose, insulin concentrations, and insulin resistance (mean ± standard deviation) in the control and experimental groups
